# Insights from clinical utility studies on rehabilitation technologies for therapy and research

**DOI:** 10.1111/dmcn.70249

**Published:** 2026-03-11

**Authors:** Hubertus J. A. van Hedel

**Affiliations:** ^1^ Research Department Swiss Children's Rehab, University Children's Hospital Zurich, University of Zurich Affoltern am Albis Switzerland

## Abstract

**This commentary is on the original article by Bradley et al. on pages** 1580–1592 **of this issue.**

Independent mobility enables children to explore their environments, engage in play, and develop relationships with peers at home, in school, and during leisure activities. Children with early‐onset disabilities such as cerebral palsy (CP) may experience mobility challenges. Powered mobility interventions can enhance not only mobility but also participation, play, and social interactions for these children.^1^ Early provision of mobility support may therefore positively influence multiple domains described by the F‐words framework.^2^ Whereas powered mobility was previously associated primarily with powered wheelchairs, recent innovations such as the Trexo now enable children with disabilities to walk and interact with peers at eye level.

Trexo was initially developed as an assistive technology to compensate for impairments and enhance the participation of children with disabilities in family and community settings. Bradley et al. have now examined its feasibility as a rehabilitation therapy device, that is, to improve function.^3^ Although the authors may have demonstrated some overenthusiasm in their small‐sample study, given the inclusion of multiple factors and diverse contexts, their work is commendable, as research involving technologies for children with severe impairments, such as 4‐ to 7‐year‐old participants in Gross Motor Function Classification System level IV, presents significant challenges.

The personalized application of new technology requires assessment of its robustness, user acceptance among therapists and patients, and its specific effects. Understanding how children with sensorimotor and cognitive impairments respond to the technology is essential for identifying potential changes in body functions. If repeated exposure to the technology results in functional improvements over time, appropriate therapeutic goals can be established. Feasibility and clinical utility studies offer preliminary insights into these considerations.

Smart identified four components of clinical utility relevant to the implementation of healthcare technology: appropriateness, practicability, acceptability, and accessibility.^4^ Bradley et al. addressed each of these components in their study.

Appropriateness encompasses the technology's effectiveness and its potential impact on the existing treatment process. The findings of Bradley et al. indicate that while certain therapeutic goals may be achieved, walking ability may not show substantial improvement. Upcoming analyses of leg muscle activation and heart rate might clarify whether Trexo increases physical activity in these children.

Practicability concerns whether the materials and methods function effectively in real‐world settings. This aspect was thoroughly evaluated, though the study environment may not fully replicate actual clinical conditions, as noted by the authors. The results indicate that the technology is safe and operates reliably, with device malfunctions being rare. Therapists and children also became 30% more efficient at donning the device over time.

Acceptability was assessed among therapists and children. While the children primarily indicated comfort and enjoyment, a previous study from this group on another technology demonstrated that the child–therapist relationship plays a critical role in acceptability,^5^ a finding we can confirm from our own clinical practice.

The final component, accessibility, encompasses procurement and maintenance costs, therapist training, and the availability, supply, and quality of the technology. The authors noted that support responses were provided within 10 minutes, with a maximum therapy delay of 15 minutes. Recently, accessibility has become increasingly important following the abrupt cessation of operations by a major international rehabilitation technology company and its service network, without prior notification to customers. Clinics utilizing rehabilitation technologies rely on the support of these companies, which must recognize their responsibilities to both rehabilitation experts and, most importantly, to young patients and their families.

Assistive technologies and rehabilitation therapy technologies offer advantages and can effectively complement conventional interventions. While Bradley et al. have provided initial information on the feasibility of Trexo as a therapeutic tool, further research is needed to investigate its efficacy and effectiveness.

## CONFLICT OF INTEREST STATEMENT

The authors declare no conflicts of interest.

## Data Availability

Not required.
